# The role of m6A demethylases in lung cancer: diagnostic and therapeutic implications

**DOI:** 10.3389/fimmu.2023.1279735

**Published:** 2023-11-29

**Authors:** Mengjiao Yu, Wenqian Ji, Xu Yang, Kai Tian, Xinyi Ma, Shali Yu, Lin Chen, Xinyuan Zhao

**Affiliations:** ^1^ Department of Occupational Medicine and Environmental Toxicology, Nantong Key Laboratory of Environmental Toxicology, School of Public Health, Nantong University, Nantong, China; ^2^ College of International Studies, Southwest University, Chongqing, China; ^3^ Nantong Institute of Liver Diseases, Nantong Third People’s Hospital Affiliated Nantong Hospital of Nantong University, Nantong, China

**Keywords:** lung cancer, m6A demethylase, FTO, ALKBH5, immunotherapy

## Abstract

m6A is the most prevalent internal modification of eukaryotic mRNA, and plays a crucial role in tumorigenesis and various other biological processes. Lung cancer is a common primary malignant tumor of the lungs, which involves multiple factors in its occurrence and progression. Currently, only the demethylases FTO and ALKBH5 have been identified as associated with m6A modification. These demethylases play a crucial role in regulating the growth and invasion of lung cancer cells by removing methyl groups, thereby influencing stability and translation efficiency of mRNA. Furthermore, they participate in essential biological signaling pathways, making them potential targets for intervention in lung cancer treatment. Here we provides an overview of the involvement of m6A demethylase in lung cancer, as well as their potential application in the diagnosis, prognosis and treatment of the disease.

## 1 Introduction

Lung cancer is a highly prevalent and devastating form of primary lung malignancy, contributing to a significant portion of cancer-related deaths worldwide. It accounts for approximately 18% of all cancer fatalities. Unfortunately, the survival rates for lung cancer remain alarmingly low, with five‐year relative survival rates of 23% (1). Understanding the diverse histopathological classifications is crucial for comprehending the complexity of this disease. In terms of histopathologically, lung cancer can be categorized into non-small cell lung cancer (NSCLC) and small cell lung cancer (SCLC). NSCLC is the most common type, and it accounts for approximately 85% of all cases, while SCLC represents about 15%. Within the subtypes of NSCLC, lung adenocarcinoma is the most prevalent histological variant, making up around 40% of NSCLC cases. Lung squamous cell carcinoma closely follows, comprising approximately 35% of NSCLC cases (2).

Epigenetic modifications play a crucial role in the initiation and progression of various tumours. These modifications mainly involve regulation of gene function and expression through processes such as DNA methylation, regulation of non-coding RNA, histone modification, and chromatin remodelling. By modifying these epigenetic marks, tumor cells can manipulate gene activity and disrupt normal cellular processes, thereby contributing to the development and advancement of cancer (3). Among the various epigenetic modifications, one of the most extensively studied is N6-adenylate methylation (m6A), which is a common internal modification in eukaryotic mRNA molecules. It represents around 60% of all known RNA modifications identified in mammals (4, 5). m6A takes place when methylation occurs at the sixth position of adenylate (A) nucleotides within the RNA molecule (6). The discovery of m6A modification provides new insights into the intricate regulation of gene expression in cancer cells. This modification serves as a dynamic and reversible mark that can impact multiple aspects of mRNA metabolism, such as splicing, stability, localization, and translation efficiency. Through m6A modification, tumor cells possess the ability to precisely regulate the expression of crucial genes that are involved in pathways essential for cell survival, proliferation, invasion, and metastasis (7). In addition, there is evidence indicating that dysregulation of m6A modification machinery and aberrant m6A patterns are commonly observed in various cancer types, can affect the occurrence and development of breast cancer, ovarian cancer, gastric cancer, lung cancer, colon cancer and other cancers (8–12). These changes can affect the behaviour of tumour cells, leading to disease advancement and resistance to treatment. Consequently, some researchers have concentrated their efforts on uncovering the specific mechanisms involved in m6A modification and its functional implications in the development of tumors (13, 14).

The occurrence of m6A modification involves the participation of multiple enzymes, including Writers, Readers and Erasers. During the transcription of DNA into RNA, the sixth N of adenosine is methylated and modified by methyltransferases such as METTL3, METTL14 and WTAP (15–17). These enzyme are referred to as Writers, specifically methylases. RNA base sites often require specific enzymes to recognize them after methylation. These enzymes, known as Readers, primarily include YTH domain proteins, nuclear heterogeneous riboproteins (hnRNPs) and eukaryotic initiation factors (eIFs) (18–20). Readers can recognize the bases where m6A methylation occurs and have the functions that include participating in mRNA degradation, downstream translation, and accelerating the rate of mRNA nuclear export. m6A demethylases are called Erasers, mainly including Fat mass and obesity-associated protein (FTO) and human AlkB homolog 5 (ALKBH5). FTO, a member of the Alkb protein family, is the first m6A demethylase that acts on mRNA in an iron-dependent manner (21). It primarily localizes in the nucleus and exhibits a punctate pattern in the nucleoplasm, partially co-localizing with splicing or splicing-related spot factor SART1 (22). Under physiological conditions, FTO demonstrates the highest affinity for m6A as a substrate. Changes or dysfunction in FTO expression may contribute to the occurrence and development of various tumors, where it can act as either a tumour suppressor gene or oncogene. FTO has been found to play a critical role in tumor cell proliferation, metastasis and apoptosis. ALKBH5, another important m6A demethylase, is capable of demethylating mRNA in the nucleus. It possesses an alanine-rich region at the N-terminus and a unique coiled-coil structure (23). Current studies have revealed dysregulation of ALKBH5 expression in various cancers, including lung, breast, and gastric cancer. ALKBH5 can exert both carcinogenic and tumor-suppressive effects, depending on the specific cancer type. Its involvement in cancer is closely related to death, migration, invasion, and metastasis (12, 24, 25).

In general, m6A demethylases catalyze the removal of methylation at the N6 position of adenylate in mRNA, thereby modulating the epigenetic information of mRNA. Upregulation of their expression decreases m6A modification, impacting the stability and translation efficiency of mRNA. m6A modification has been implicated in the promotion of lung cancer growth and progression, affecting the proliferation, invasion, and metastasis of lung cancer cells. Aberrant m6A modification may result from abnormal expression of writers and erasers. When exposed to external factors such as environmental pollutants, m6A modification abnormalities may occur, influencing the onset and progression of lung cancer and other cancers. Additionally, other proteins and signaling pathways involved in m6A modification may also play a role in lung cancer. m6A modification can influence the characteristics and maintenance of lung cancer stem cells, thereby facilitating tumor initiation (26, 27). It can also impact the transcription and translation processes in lung cancer cells, thereby regulating key signaling pathways and promoting tumor progression and metastasis. Moreover, m6A modification can modulate the expression of genes associated with lung cancer by altering mRNA stability, thereby affecting crucial processes such as cell cycle control and apoptosis (10, 28). Notably, lung cancer is a multifaceted disease influenced by various contributors, including genetic mutations, epigenetic alterations, and environmental factors. Thus, further investigations are needed to comprehensively elucidate the precise mechanisms underlying m6A modification in lung cancer.

**Figure 1:**
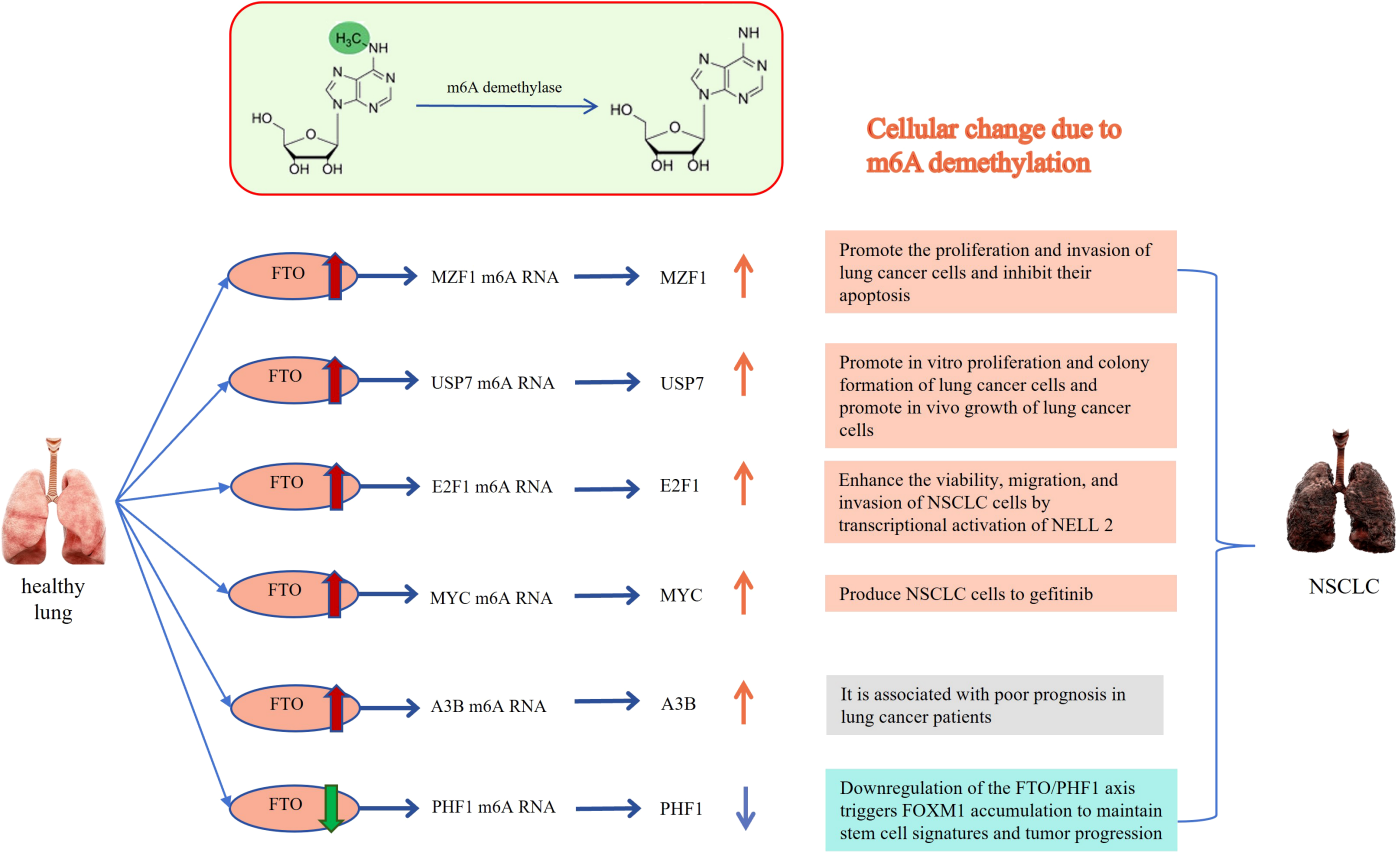
Relationship between FTO and lung cancer: The m6A methylated RNA can be demethylated by FTO, resulting in the occurrence of cytological behaviors that contribute to the development of NSCLC. MZF1, Myeloid Zinc Finger Protein 1; USP7, ubiquitin-specific protease-7; E2F1, E2F transcription factor-1; MYC, v-myc avian myelocytomatosis viral oncogene homolog; A3B, APOBEC3B; PHF1, Human Plant Homeodomain (PHD) finger protein 1. The Red and orange arrows represent increased m6A demethylase levels or m6A modification levels of RNA; The green and blue arrows represent decreased m6A demethylase levels or m6A modification levels of RNA.

**Figure 2:**
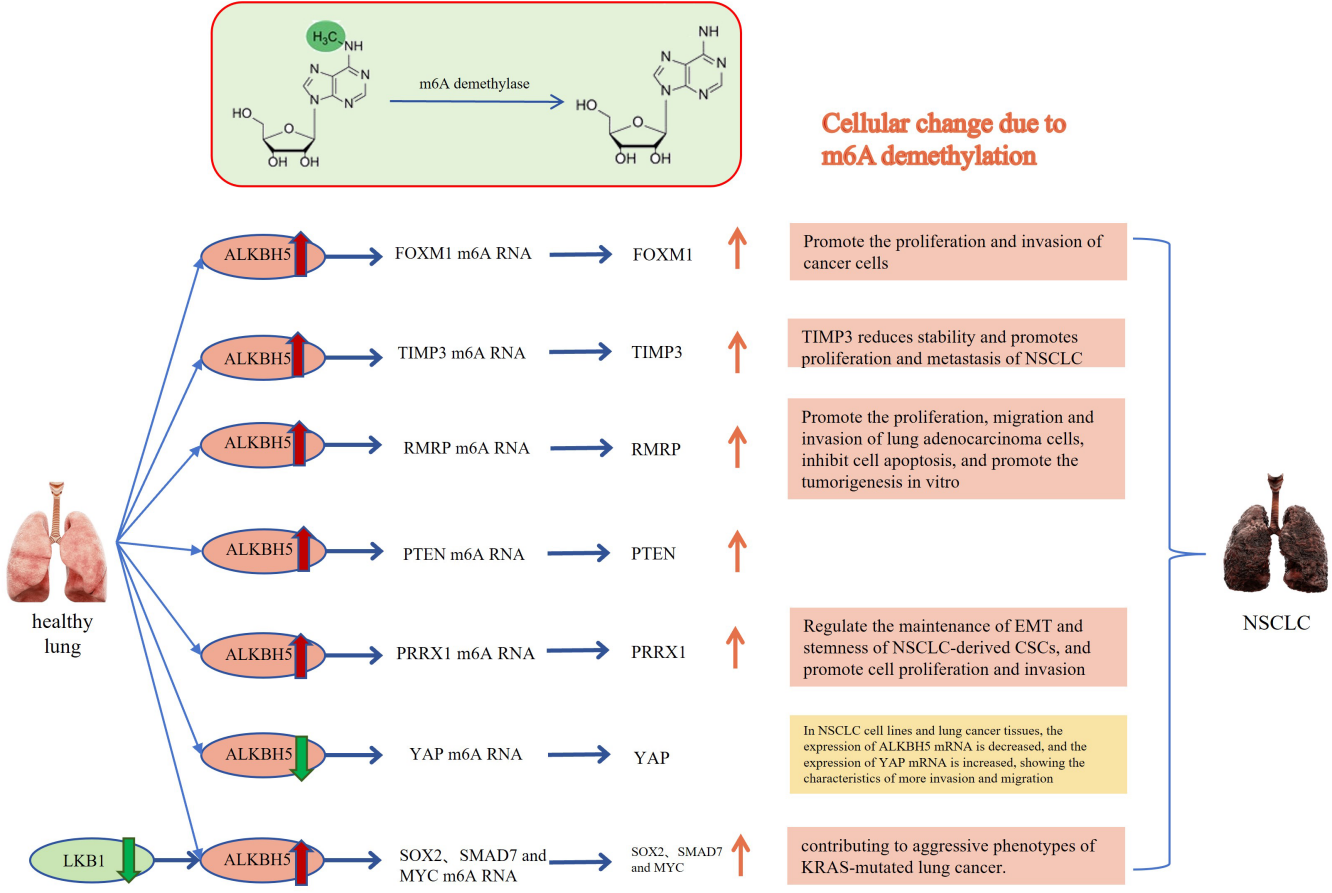
Relationship between ALKBH5 and lung cancer: The m6A methylated RNA undergoes demethylation through the action of ALKBH5, leading to the occurrence of cytological behaviors that promote the development of NSCLC. FOXM1, Forkhead box M1; TIMP3, TIMP metallopeptidase inhibitor 3; RMRP, RNA Component of Mitochondrial RNA Processing Endoribonuclease; PTEN, Phosphatase and tensin homolog deleted on chromosome 10; PRRX1, Paired related homeobox 1; YAP, Yes-associated protein; SOX2, SRY-box transcription factor 2; SMAD7, SMAD family member 7. The Red and orange arrows represent increased m6A demethylase levels or m6A modification levels of RNA; The green and blue arrows represent decreased m6A demethylase levels or m6A modification levels of RNA.

Understanding the significance of m6A modification in cancer is crucial for the advancement of targeted treatment strategies and the discovery of potential biomarkers. By unraveling the intricate interplay between m6A writers, erasers, and readers, researchers can gain insights into the molecular mechanisms driving tumor progression. Recent studies have highlighted the importance of FTO and ALKBH5, as erasers involved in m6A RNA demethylation, in the development of lung cancer. These demethylases have been found to be closely associated with occurrence and development of the cancer. Furthermore, we will discuss future directions of research in this field and explore the potential clinical applications of targeting of FTO and ALKBH5 in the treatment of lung cancer. The relationship between erasers and lung cancer is shown in **Figures 1**, **2**.

## 2 The impact of m6A demethylases on lung cancer progression

m6A, as the most prevalent mRNA modification, has methyltransferases like METTL3 being responsible for adding methyl groups to specific mRNA sites, while m6A demethylases FTO and ALKBH5 are responsible for removing methyl groups from m6A. The interaction between methyltransferases and demethylases helps maintain a balanced level of m6A. When m6A levels are stable, mRNA transcripts with appropriate m6A levels can undergo proper splicing, transport, translation, or degradation. Imbalanced m6A regulation can lead to RNA metabolism defects (29, 30). FTO and ALKBH5 are known to be upregulated in lung cancer tissues. They have been found to play a crucial in tumor cell proliferation, apoptosis, invasion, and migration, and their functions are dependent on the presence of m6A modifications (31, 32). These demethylases also contribute to the regulation of tumor progression in lung cancer (33). The possible mechanisms by which m6A demethylation affects the development and progression of lung cancer are listed in **Table 1**.

**Table 1:** Biological function of M6A demethylase in lung cancer.

Function	m6A demethylases	Targeted genes	Ref.
Involved in regulating the proliferation and invasion of lung cancer cells.	FTO, ALKBH5	USP7, E2F1	(34–36)
Regulate angiogenesis in the lungs.	ALKBH5	PVT1	(37)
Regulate transcriptional and post-transcriptional modification levels in lung cancer cells.	FTO, ALKBH5	MZF1	(38, 39)
Affect drug tolerance of lung cancer cells.	FTO, ALKBH5	ABCC10, cMyc, WIF1	(40–42)
Regulate the properties and self-renewal ability of lung cancer stem cells.	FTO, ALKBH5	KRT7	(26, 27)
Participate in the metastasis and invasion process of lung cancer.	FTO, ALKBH5	UBE2C, IGF2BP	(27, 28)
Affect the mechanism of apoptosis and apoptosis evasion in lung cancer cells.	FTO, ALKBH5	UBE2C, TIMP3, IGF2BP	(10, 28, 43)
Interact with other signaling pathways to affect the survival and proliferation of lung cancer cells	FTO, ALKBH5	FOXM1, miR-607, A3B	(24, 44, 45)

### 2.1 The role of demethylase overexpression in cell proliferation and apoptosis

The research on the relationship between demethylase and lung cancer, as well as its mechanism is still in the early stages. However, increasing evidence suggests that m6A demethylases FTO and ALKBH5 are frequently overexpressed in lung cancer and significantly associated with the tumor’s prognosis. As research progresses, the involvement of m6A modification in lung cancer is gradually being confirmed. Numerous studies have demonstrated that the demethylation process in lung cancer cells plays a crucial role in regulating the expression of key genes involved in essential cellular processes such as cell proliferation and apoptosis. Disturbances in DNA methylation patterns can disrupt the normal regulation of these genes, leading to increased proliferation of lung cancer cells and decreased apoptosis. These alterations contribute to progression and aggressiveness of tumors (46, 47). In lung cancer, abnormal DNA demethylation can occur at specific genomic regions, resulting in the activation of oncogenes or the silencing of tumor suppressor genes (48). Through the removal of the methyl groups from the DNA molecule, demethylation can lead to the overexpression of genes that promote cell proliferation, giving cancer cells a growth advantage (10). Moreover, the suppression of genes responsible for apoptosis can enable cancer cells to evade programmed cell death, aiding in their survival and expansion (28).

Li et al. conducted a study that revealed a significant association between high expression of m6A demethylase FTO and cancer development. The overexpression of FTO could promote cell proliferation and colony formation (34, 49, 50). Both mRNA and protein levels of FTO were observed to be overexpressed in human NSCLC tissues and cell lines. Conversely, the loss of FTO function resulted in a reduced proliferation rate of cancer cells. Mechanistically, FTO can decrease m6A levels through its demethylase activity, enhance the stability of ubiquitin-specific protease (USP7) mRNA, and promote the growth of lung cancer cells (49, 50). Another study demonstrated that FTO can promote the proliferation and invasion of LUSC by reducing the m6A level in myeloid zinc finger 1 (zinc finger 1, MZF1) mRNA transcription and maintaining the mRNA stability (38). Additionally, some studies have shown that circular RNAs can act as miRNA sponges, promoting cancer cell proliferation and participating in the process of NSCLC. For instance, Hsa_circ_0072309 can sponge miR-607, leading to the upregulation of its target gene FTO and promoting the occurrence of NSCLC (44). Another m6A demethylase also ALKBH5 also plays a key role in promoting or inhibiting cell proliferation and apoptosis. FOXM1 is involved in important cellular processes such as cell proliferation, cell differentiation, cell cycle regulation, angiogenesis and metastasis. It is often upregulated in human malignant tumors, indicating a poor prognosis (51, 52). Studies have shown that upregulation of ALKBH5 in cancer cells can enhance the translation efficiency of FOXM1 mRNA, leading to increased FOXM1 protein expression and promoting the proliferation and invasion of cancer cells (24). Similarly, Zhu et al. demonstrated in their studies that ALKBH 5 promotes the proliferation of NSCLC cells and reduces apoptosis *in vitro*. Knockdown of ALKBH5 *in vivo* inhibited tumor growth primarily by destabilizing TIMP 3 mRNA. ALKBH5 interacts with the methylation site of TIMP3 in the 3’UTR to facilitate protein production (10). Additionally, studies have shown that knockout ALKBH5 can induce G1 phase arrest, inhibit the cancer cell proliferation, and increase the number of apoptotic cells (43). ALKBH5 also can indirectly regulating autophagy. For instance, UBE2C, a ubiquitin-conjugating enzyme, selectively inhibits autophagy in NSCLC. Destroying UBE2C-mediated autophagy inhibition weakens the proliferation and clonogenicity of NSCLC. ALKBH5 stabilizes UBE2C transcription by reducing the m6A methylation level in its mRNA (28). Indeed, m6A demethylation plays a significant role in lung cancer cell proliferation, providing important insights into the understanding and treatment of lung cancer. The biological function and specific mechanism leading to cancer development and progression of m6A demethylation are not fully understood, and further research is required for validation.

### 2.2 m6A demethylases-mediated regulation of invasion and migration in lung cancer cells

Demethylation is closely related to the invasion and migration of lung cancer cells as well. Studies have shown that demethylation can affect the metastatic ability of lung cancer cells, thus facilitating the metastasis and invasion of these cells. Demethylation plays a role in gene expression regulation, particularly in relation to cell metastasis by transcription factors and signaling molecules. One example is the activation of cell migration by FTO through mRNA demethylation. This activation can contribute to the progression of lung cancer and affect the prognosis of patients (35). Liu et al.’s study demonstrated that the higher levels of FTO are associated with worse prognosis in LUSC patients. FTO acts as an m6A mRNA demethylase, promoting cell migration and invasion (38). Another study found that FTO can inhibit the M6A modification of E2F1 *in vivo*, leading to increased expression of E2F1. E2F1, in turn, enhances the survival, migration, and invasion of NSCLC cells by activating NELL2 transcriptionally. Furthermore, FTO can promote the formation and metastasis of NSCLC through the FTO/E2F1/NELL2 axis *in vitro* (36). As for ALKBH5, some studies have found that ALKBH5 can promote cell migration and invasion. In lung cancer patients, the levels of ALKBH5 protein expression have been observed to be positively associated with tumor size, TNM staging, and clinical staging. Research by Yu et al. indicated that patients with higher ALKBH5 gene expression had a poorer prognosis (53). ALKBH5 upregulates the expression of RMRP through demethylation, and the upregulation of RMRP promotes the proliferation and invasion of lung adenocarcinoma cells while inhibiting cell apoptosis. However, other researchers have discovered that overexpression of ALKBH5 effectively reverses the proliferation, colony formation and migration of kras mutant lung cancer cells that are regulated by LKB1 (47).

The impact of demethylation on the phenotypic transition of cells, such as the transition from epithelial cells to mesenchymal cells, and its effect on the invasion and migration of lung cancer cells have yielded different conclusions, including both promotion and inhibition of lung cancer cell migration by ALKBH. Experiments conducted by Guo et al. demonstrated that downregulation of ALKBH5 using miR-381 and siRNA specifically impedes the EMT of NSCLC cells, thereby inhibiting their migration and invasive growth (28). Liu et al. also found that ALKBH 5 is highly expressed in NSCLC-derived CSCs (cancer stem cells), and downregulation of ALKBH5 significantly reduces Sox2 expression, leading to increased levels of E-cadherin protein, inhibition of EMT, and suppression of tumor invasive development and metastasis (54). However, some researchers have come to the opposite conclusion. Studies have shown that TGF-β induces EMT in NSCLC and regulate cell migration and invasion. Interestingly, overexpression of ALKBH 5 can inhibit the metastasis of NSCLC cells stimulated by TGF-β *in vivo* (45). Jin et al. also believe that ALKBH5 acts as an inhibitor of NSCLC cell migration and invasion, ALKBH5 regulates the miR-107/LATS2 axis in a HURP-dependent manner, thereby reducing the expression and activity of YAP, thereby inhibiting tumor growth and metastasis (48). The possible explanation for this phenomenon is that when the gene interacts with a tumor suppressor gene it promotes cancer cell growth and metastasis while when it interacts with an oncogene it inhibits cancer cell growth. In addition, lung cancer is a highly heterogeneous disease, and biological diversity and subtype differences in lung cancer need to be considered. The specific reasons for the differences still need to be explored. Notably, patients with Trp53 gene mutations tend to have a poorer prognosis when FTO is expressed at high levels, while patients with the wild-type gene don’t exhibit the same trend (39). This suggests that the observed phenomenon may be associated with specific genotype mutations, highlighting the need for further research. Overall, demethylation is a critical factor in the invasion and migration of lung cancer cells, playing a role in their metastatic potential. By influencing gene expression, the phenotypic transition of cells and other processes, demethylation can promote the acquisition of invasive and migratory characteristics in lung cancer cells. Further research focusing on the identification of specific genes and molecular pathways affected by demethylation in the context of lung cancer metastasis could offer valuable insights for targeted therapies to address metastatic disease.

## 3 Potential applications of m6A demethylase in the diagnosis, prognosis assessment and treatment of lung cancer

### 3.1 Application of m6A demethylase in the lung cancer diagnosis

Recent studies have shown that significant application value of m6A demethylase in diagnosing lung cancer. Specifically, by comparing the expression levels of m6A demethylase in lung cancer tissue and normal lung tissue, researchers have observed significant alterations in its expression, either upregulation or downregulation, indicating its potential as a diagnostic marker for lung cancer. These findings have garnered considerable attention in the field of lung cancer diagnosis. Among the m6A demethylases, FTO and ALKBH5 may be associated with lung cancer, and their relationship has been gradually studied. These enzymes have demonstrated potential in developing risk assessment models that enhance the accuracy of diagnosing lung adenocarcinoma (LUAD), a prevalent histological subtype of lung cancer. By incorporating methylation-related enzymes, such as FTO and ALKBH5, into these risk assessment models, researchers have achieved improved prediction accuracy for LUAD diagnosis (55). Moreover, evaluating the expression levels of m6A demethylases in blood or tissue samples from lung cancer patients holds the potential for early diagnosis, pathological classification, and staging of lung cancer staging (47). The application of m6A demethylases in lung cancer diagnosis is an exciting area of research with significant clinical implications. By incorporating these enzymes into diagnostic algorithms and utilizing their association with tumorigenesis, healthcare professionals may enhance the accuracy and efficiency of lung cancer diagnosis (56). Additionally, the ability to detect m6A demethylase expression in various sample types, including blood and tissue, may offer a non-invasive and easily accessible approach for the early detection and monitoring of lung cancer (57, 58). Indeed, additional research is required to validate and enhance the diagnostic potential of m6A demethylases in lung cancer. Large-scale clinical studies involving diverse patient populations are necessary to establish robust diagnostic models and determine the specific thresholds for demethylase expression levels in different stages and subtypes of lung cancer (59, 60). These investigations will aid in developing standardized diagnostic protocols and facilitate the integration of m6A demethylases into routine clinical practice for accurate lung cancer diagnosis. In summary, recent studies have demonstrated the potential value of m6A demethylases in diagnosing lung cancer. The differential expression of these enzymes in lung cancer tissue and their potential integration into risk assessment models underscore their diagnostic potential (61). Moreover, assessing m6A demethylase expression levels in blood or tissue samples shows promise for early diagnosis, pathological typing, and lung cancer staging (62). Further research and validation are needed to fully realize the diagnostic capabilities of m6A demethylases, leading to improved lung cancer diagnosis and patient care.

### 3.2 Application of m6A demethylase in the lung cancer prognosis assessment

m6A demethylases have shown significant potential in the prognostic assessment of lung cancer. The expression levels of these enzymes in lung cancer tissues have been strongly correlated with patient survival rates and overall prognosis (63). Abnormally expression of demethylases, either too high or too low, may serve as indicators of poor clinical outcomes. Studies have indeed reported that high expression of ALKBH5 and FTO is associated with a favourable prognosis in lung cancer patients (64). Elevated levels of these demethylases may indicate better outcomes for patient (65). Furthermore, the expression levels of m6A demethylases can be used to develop prognostic models to assess the risk of poor prognosis in patients. Recent research has focused on the construction of such models using factors including FTO and other genes. For instance, Zhang et al. developed a model, and found that a two-gene model combining FTO and METTL3 was more effective in guiding prognostic assessment of lung cancer (66). Additionally, researchers have utilized methylation-related enzymes such as FTO and ALKBH5 to develop risk assessment models for LUAD patients. These models divide LUAD patients into high-risk and low-risk categories based on the constructed models, and correlations have been observed with various clinical factors including TNM staging, lymph node staging, gender, and tumour stage (55). This suggests that the risk assessment model based on m6A demethylases can provide valuable insights into the prognosis and clinical characteristics of LUAD patients. These findings highlight the potential of m6A demethylases as prognostic markers in lung cancer. Incorporating m6A demethylase expression levels into prognostic models or risk assessment models can improve the accuracy of prognostic evaluation and assist in clinical decision-making. By considering the expression levels of these demethylases along with other relevant clinical factors, healthcare professionals can better predict patient outcomes and tailor treatment strategies accordingly. It is important to note that further research and validation are necessary to establish standardized prognostic models incorporating m6A demethylases in lung cancer (67, 68). Large-scale studies involving diverse patient populations must be conducted to confirm the associations between demethylase expression, prognostic risk, and clinical characteristics (69). These efforts will contribute to developing reliable prognostic tools that can guide patient management and improve prognostic assessments in lung cancer. m6A demethylase, along with other m6A regulatory factors, collaborates to regulate methylation in the body. Relying solely on ALKBH5 and FTO is not reliable for diagnosing and predicting prognosis in lung cancer patients. Therefore, it is more meaningful to collectively detect various regulatory factors than focusing on individual ones, as it can better reflect the actual conditions of lung cancer patients (70, 71). In clinical practice, monitoring the expression level of m6A demethylase in lung cancer patients is expected to offer personalized treatment recommendations and an accurate assessment of efficacy and prognosis. Numerous studies have indicated that m6A demethylase has the potential as a diagnostic and prognostic marker for lung cancer.

### 3.3 Application of m6A demethylation drugs in lung cancer treatment

Lung cancer, particularly NSCLC, continues to be the primary cause of cancer-associated mortality globally, constituting 85% of newly diagnosed cases (72). With the in-depth study of m6A demethylase mechanism in lung cancer, the potential application of m6A demethylation drugs in treating lung cancer is becoming increasingly evident. Significant progress has been made in the laboratory and clinical trials small molecule inhibitors and activators targeting demethylases such as FTO and ALKBH5 have made certain research progress. At present, the most commonly used m6A demethylase drugs are mainly focused on m6A demethylase inhibitors, which can regulate the activity of m6A demethylase by binding to its active site. FTO inhibitors included FB23 and FB23-2, rhein, Meclofenamic acid, fluorescein, R-2-hydroxyglutarate (R-2HG), et al. (73–77). In recent years, 44/ZLD115, Xanthine derivatives and other FTO inhibitors have been developed. Among them, 44/ZLD115 shows good anti-leukemia activity in xenograft mice and is a very promising FTO inhibitor (78, 79). With the continuous progress of scientific research, the development of ALKBH inhibitors is also ongoing. For example,2- [(1-hydroxy-2-oxo-2-phenylethyl) sulfanyl] acetic acid (3) and 4-[(furan-2-yl) methyl] amino-1,2-diazinane-3,6-dione (6). These two novel ALKBH5 inhibitors could selectively inhibit the growth of leukemia cell lines (80). These drugs have shown promise in affecting tumor cell growth, invasion and metastasis by regulating the activity of m6A demethylase. However, the current development of m6A demethylase inhibitors is mainly focused on various types of leukemia, breast cancer, bowel cancer and other cancers, but they have shown promising characteristics, while m6A demethylase inhibitors for lung cancer still need to be developed by researchers.

A research team reported that the synthesis of an FTO inhibitor, which demonstrated its ability to inhibit cancer progression via the inhibition of cell invasion, migration, and EMT (epithelial-mesenchymal transition). Additionally, the inhibitor showed potential in inhibiting angiogenesis, a critical process for tumor growth and metastasis (27). Therapeutic resistance to multiple small molecules, including chemotherapeutics and targeted agents, is a significant factor contributing to poor prognosis in NSCLC (71, 81). Gefitinib, an essential drugs for treating lung cancer, has been found to be associated with demethylase-related resistance in NSCLC. Specifically, studies have reported that there is an association between the methylation level of WIF1 in cfDNA (Cell-free DNA) and the insensitivity of gefitinib in the treatment of lung cancer. In patients with more advanced disease, the DNA methylation levels of the WIF1 promoter are significantly elevated (41). Co-administration of GE (gefitinib) with MA (meclofenamic acid) has been demonstrated to enhance the sensitivity of drug-resistant NSCLC cells to treatment (42). This effect is attributed to the inhibition of BCRP and MRP7 expression levels through the FTO/m6A/MYC axis. These findings suggest that combining drugs can potentially overcome treatment resistance in NSCLC. Moreover, exosomes derived from gefitinib-resistant (GR) cells can play a role in intercellular transmission of gefitinib resistant through the FTO/YTHDF2/ABCC10 axis. These findings confirm the Feasibility of targeting FTO-m6A axis to prevent or delay the acquisition of gefitinib resistance in NSCLC (40).

In the future, m6A demethylation drugs have the potential to become a vital component of the comprehensive treatment strategy for lung cancer. By combining these drugs with traditional treatments such as surgery, radiotherapy, and chemotherapy, it is expected that the survival rate and quality of life for lung cancer patients can be improved. It is important to highlight that the research and development of drugs targeting m6A demethylation still encounter certain challenges. Firstly, there is a need to enhance the effectiveness of these drugs and minimize the occurrence of side effects by further refining their the selectivity and specificity (82). In addition, owing to the considerable heterogeneity of lung cancer, variations in the response of different patients to m6A demethylating drugs may exist (83). Hence, it is imperative to study biomarkers that can predict patient response to drugs and prognosis in order to achieve precision medicine (84). Overall, m6A demethylases hold significant potential for applications in lung cancer diagnosis, prognosis assessment, and therapy. Future research should focus on gaining a deeper understanding of the mechanism of action of m6A demethylase, expediting the development of drugs, and providing more effective and personalized treatment options for lung cancer patients (85). Additionally, interdisciplinary cooperation and clinical trials will play an essential role in advancing the application of M6A demethylase in lung cancer.

## 4 Environment specific factor for abnormal m6A modification in lung cancer

Research has indicated that prolonged exposure to environments with heavy metals increases the risk of lung cancer in humans. For instance, exposure to beryllium (Be) and arsenic (As) compounds, both *in vivo* and *in vitro* is strongly associated with the occurrence and development of lung cancer (86). m6A demethylase also plays a role in the interaction between heavy metals and lung cancer. For example, FTO protein is highly expressed in tumor samples of NSCLC patients and it can mediate the reduction of m6A modification induced by arsenic in A3B, resulting in increased expression of A3B (87). Similarly, ALKBH5 can regulate the m6A methylation level of PTEN mRNA and reduce the stability of PTEN mRNA and promoting the cadmium-induced malignant transformation of human bronchial epithelial cells, as well as enhancing proliferation, migration and invasion of cancer cells (88). Furthermore, ALKBH5 may be involved in silica-induced pulmonary fibrosis, which may be through the miR-320a-3p/FOXM1 axis or by directly targeting FOXM1 (89). However, there is limited knowledge regarding how m6A demethylases contribute to the pathogenesis of lung cancer and their relationship with heavy metal exposure. Studying and understanding these mechanisms is of great significance as it can provide new ideas and methods for the treatment and prevention lung cancer.

## 5 Discussion

In recent years, m6A demethylase, a novel RNA-modifying enzyme, has emerged as a prominent molecular mechanisms in lung cancer research. Studies have demonstrated that m6A demethylase affects the stability, transcription, and translation of target genes, subsequently impacting the proliferation, migration, invasion and other processes of lung cancer cells. It also interacts with other molecules such as MZF1, USP7, FOXM1, forming a complex molecular regulatory network. Additionally, m6A demethylase can regulate certain MicroRNAs, including miR-107, miR-607 (44, 48), thereby affecting various physiological functions in the body. Furthermore, researchers have discovered that m6A demethylase may also have an impact on cancer metastasis. For example, m6A modification can regulate the malignancy of breast cancer lung metastasis cells, and overexpression of FTO can significantly inhibit the lung colonization of BT-549LMF3 cells (26). All these findings highlight the close association between m6A demethylase and the malignant progression of lung cancer. Increasing evidence suggests that m6A demethylase holds significant potential in the diagnosis, prognosis evaluation, and treatment of lung cancer. However, the current research is unable to fully elucidate the complete functionality of m6A demethylase. m6A methylation can be likened to a double-edged sword since its overexpression may contribute to certain tumor types, while the absence of m6A modification may drive the progression of other tumors. The inconsistent results observed among researchers can be attributed to various factors. Therefore, conducting more multicenter, large-scale studies is imperative in order to delve deeper into this topic and establish a solid foundation for the effective treatment of human tumors. Advancing the field of m6A demethylases requires a thorough investigation into their underlying mechanisms. By unravelling the complex molecular pathways and regulatory networks involved in the malignant transformation of lung cancer cells, researchers can lay the foundation for development of more precise and targeted treatments (90). Gaining a comprehensive understanding of the specific role of m6A demethylases within these networks will provide valuable theoretical insights and offer practical support for the design and implementation of effective therapeutic strategies. Additionally, investigating molecular network regulation models involving m6A demethylases in lung cancer holds great potential. By unraveling the intricate interactions between m6A demethylases and other critical genes implicated in the progression of lung cancer, researchers can identify novel targets and pathways that can be targeted for therapeutic purposes (91, 92). This approach has the potential to revolutionize the treatment landscape by enabling the development of innovative combination therapies or the identification of specific molecular signatures that can guide personalized treatment approaches for individuals with lung cancer.

Although it will be some time before m6A demethylase can be applied to clinical practice, the current study has shown its translational value. This requires large-scale clinical samples and cell model studies to obtain sufficient data support. In addition, accurate and meticulous analysis of the large amount of RNA modification data is also a difficult point that needs to be broken through. The development of targeted drugs for different stages of lung cancer may be challenging and promising.

## 6 Conclusion

Overall, m6A modification is one of the most common RNA modifications. It not only plays an important role in various cell biological processes, but also participates in the occurrence and development of cancers, such as lung cancer, acute myeloid leukemia and breast cancer, etc. Therefore, m6A-modified related molecules are considered as potential tumor diagnostic markers and therapeutic targets. Conducting in-depth research on the mechanisms of m6A demethylase function and its intricate network interactions in lung cancer will greatly enhance our understanding of the disease and o provide opportunities for targeted treatments. Through rigorous scientific inquiry, researchers can establish the groundwork for the development of more effective therapies to fight against lung cancer and well-being of patients affected by this condition.

## Author contributions

MY: Methodology, Conceptualization, Data curation, Formal Analysis, Investigation, Writing – original draft. WJ: Methodology, Supervision, Writing – review & editing. XY: Data curation, Formal Analysis, Writing – review & editing. KT: Writing – review & editing, Formal Analysis, Visualization. XM: Formal Analysis, Visualization, Writing – review & editing. SY: Writing – review & editing, Conceptualization, Supervision. LC: Conceptualization, Supervision, Writing – review & editing. XZ: Supervision, Writing – review & editing, Funding acquisition, Methodology, Project administration.
